# Genetic profiling of a rare condition: co-occurrence of albinism and multiple primary melanoma in a caucasian family

**DOI:** 10.18632/oncotarget.12777

**Published:** 2016-10-20

**Authors:** Simona De Summa, Michele Guida, Stefania Tommasi, Sabino Strippoli, Cristina Pellegrini, Maria Concetta Fargnoli, Brunella Pilato, Iole Natalicchio, Gabriella Guida, Rosamaria Pinto

**Affiliations:** ^1^ IRCCS Istituto Tumori “Giovanni Paolo II”, Molecular Genetics Laboratory, Bari, Italy; ^2^ IRCCS Istituto Tumori “Giovanni Paolo II”, Oncology Unit, Bari, Italy; ^3^ University of L’Aquila, Department of Biotechnological and Applied Clinical Sciences, L’Aquila, Italy; ^4^ Section of Clinic Pathology, OO.RR., Foggia, Italy; ^5^ University of Bari, Department of Medical Biochemistry, Bari, Italy

**Keywords:** multiple primary melanoma, albinism, MGMT, family study, susceptibility

## Abstract

Multiple primary melanoma (MPM) is a rare condition, whose genetic basis has not yet been clarified. Only 8-12% of MPM are due to germline mutations of CDKN2A. However, other genes (POT1, BRCA1/2, MC1R, MGMT) have been demonstrated to be involved in predisposition to this pathology.

To our knowledge, this is the first family study based on two siblings with the rare coexistence of MPM and oculocutaneous albinism (OCA), an autosomal recessive disease characterized by the absence or decrease in pigmentation in the skin, hair, and eyes.

In this study, we evaluated genes involved in melanoma predisposition (CDKN2A, CDK4, MC1R, MITF, POT1, RB1, MGMT, BRCA1, BRCA2), pathogenesis (BRAF, NRAS, PIK3CA, KIT, PTEN), skin/hair pigmentation (MC1R, MITF) and in immune pathways (CTLA4) to individuate alterations able to explain the rare onset of MPM and OCA in indexes and the transmission in their pedigree.

From the analysis of the pedigree, we were able to identify a “protective” haplotype with respect to MPM, including MGMT p.I174V alteration. The second generation offspring is under strict follow up as some of them have a higher risk of developing MPM according to our model.

## INTRODUCTION

The occurrence of multiple primary melanoma (MPM) is quite rare, with an estimated incidence ranging from 0.2%-8.6% [1]. A genetic component in the pathogenesis of MPM is suggested by the evidence that patients who have already been diagnosed with one primary melanoma have a substantially increased risk of developing further primary melanomas, ranging from 0.6% to 12.7%. The most important risk factors associated with the development of MPM seem to be the positive family history of melanoma and the presence of atypical nevi [2].

Melanoma predisposition is related to germline mutations in several high and medium penetrance genes. CDKN2A gene mutations have been widely reported as the most common cause of inherited susceptibility to melanoma [3]. An increase in the frequency of CDKN2A mutations was observed in patients whose relatives had MPM. In fact, CDKN2A germline mutations have been identified in less than 2% of single primary melanoma cases, 8-12% of sporadic MPM cases and 47% of MPM patients with familial melanoma (fM) [4,5]. p16INK4A is produced from the transcript of exons 1a, 2 and 3 of the CDKN2A gene. The main tumour suppressor activity of p16INK4A is exploited through inhibition of CDK4 and CDK6, thus maintaining retinoblastoma protein (RB1) in a hypophosphorylated state to prevent cell cycle S-phase entry.

Although about 50% of the fM cases might be attributed to variants in CDKN2A and CDK4, a larger number of genes should be investigated.

Recent studies, performed by two different groups [6,7], identified rare germline variants of POT1 in fM cases negative for mutations in CDKN2A and CDK4.

MGMT is a crucial gene in DNA repair pathways, and its loss is also associated with an increased risk of melanoma development [8].

MC1R and MITF are considered moderate-risk genes for melanoma susceptibility [9–11]. The p.R151C, p.R160W and p.D294H variants in MC1R and the p.E318K in MITF were reported to be associated with the risk of cutaneous cancer [10,12]. A recent study showed that MITF is responsible for sporadic MPM susceptibility in about 3% of cases [13]. Moreover, MC1R and MITF, other than being moderate-penetrance melanoma susceptibility genes, play an important role in the pigmentation of skin and hairs.

The association between melanoma and BRCA1/2 mutations remains an open question which needs clarifications. BRCA1 mutations have not been significantly associated with melanoma. In contrast, suspected BRCA2 mutation carriers have shown an increased risk (2-5 times) of developing melanoma compared with the general population [14].

The RAS/RAF/MEK/ERK and PI3K/AKT signalling pathways are strongly involved in the pathogenesis of cutaneous melanoma (CM). The RAS/RAF/MEK/ERK pathway, activated in >80% of all CM by BRAF or NRAS mutations, has been shown to promote cell proliferation, cell survival and tumor metastasis. The inactivation of PTEN is present in 40–60% of sporadic melanomas, which in turn negatively regulates the PI3K/AKT pathway conferring invasive and stem cell like features to melanoma cells.

The study of immune checkpoints is another interesting field in melanoma research. CTLA4 is expressed on T cells acting as a negative regulator and inhibiting host immune response to melanoma. Furthermore, an association between CTLA4 gene polymorphisms and skin autoimmune disease like vitiligo has been firmly established [15] as well as with non melanoma skin cancer. However, RAS/RAF/MEK/ERK, PI3K/AKT and immune signalling pathways are scarcely explored in MPM pathogenesis.

To our knowledge, this is the first family study based on two siblings with the rare coexistence of MPM and oculocutaneous albinism (OCA), an autosomal recessive disease characterized by the absence or decrease in pigmentation in the skin, hair, and eyes. OCA patients have a higher incidence of squamous cell carcinomas than the general population, but melanomas are rare and usually amelanotic.

In this study, we evaluated genes involved in melanoma predisposition (CDKN2A, CDK4, MC1R, MITF, POT1, RB1, MGMT, BRCA1, BRCA2), pathogenesis (BRAF, NRAS, PIK3CA, KIT, PTEN), skin/hair pigmentation (MC1R, MITF) and in immune pathways (CTLA4) to individuate alterations able to explain the rare onset of MPM and OCA in indexes. Moreover, differences with three other siblings and the possible transmission of these alterations to their relatives have been analyzed.

## RESULTS

### Mutational study

Ten nanograms of DNA were processed according to the manufacturer’s protocol. In all samples an adequate library for subsequent sequencing was obtained, and at least one alteration of the genes included in our Next Generation Sequencing (NGS) custom panel was shown in each analyzed specimen.

Variant calling was carried out by the variant caller of Torrent Suite and a custom pipeline (see Materials and Methods section). The custom pipeline showed 89% and 94% sensitivity and specificity respectively for single nucleotide variant calling. Thus, we considered variants called by both methods. Regarding indels, hard filtering of our custom pipeline needs improvement given the high discordance rate.

Of the custom panel genes, three of them (NRAS, PTEN and CDK4) did not reveal any type of alterations. The most altered genes resulted MC1R (94%), CTLA4 (65%), MGMT and PIK3CA (both 53%). In fact, all samples except one showed MC1R missense variants classified as **s**trong RHC (p.Ile155Thr, p.Arg142His, p.Asp294His) or weak RHC (p.Val60Leu) variants. Moreover, in one sample, a not yet classified MC1R variant was found (p.Phe235Leu). All CTLA4 altered cases showed p.Thr17Ala SNP. Instead, the PIK3CA alterations more frequently individuated were p.Glu707Lys and p.Ile391Met.

As regards the BRAF gene, we did not report any mutations, but only a synonymous variant (p.Gly643=). Furthermore, in this study, MITF, KIT and RB1 resulted to be genes with few variants yet to be classified.

Interestingly, when we consider the indexes and their three healthy siblings, it is possible to note that the MC1R genetic pattern, including the missense variant p.Ile155Thr and the synonymous alteration p.Thr314=, is present in all 4 siblings with OCA, explaining the feasible relation with albinism. On the contrary, the indexes did not show MGMT p.Ile174Val, found in the other three healthy siblings, probably demonstrating that the absence of this alteration may be related to the onset of melanoma. BRCA1/2 variants were searched in the two indexes finding no mutation.

**Figure 1:**
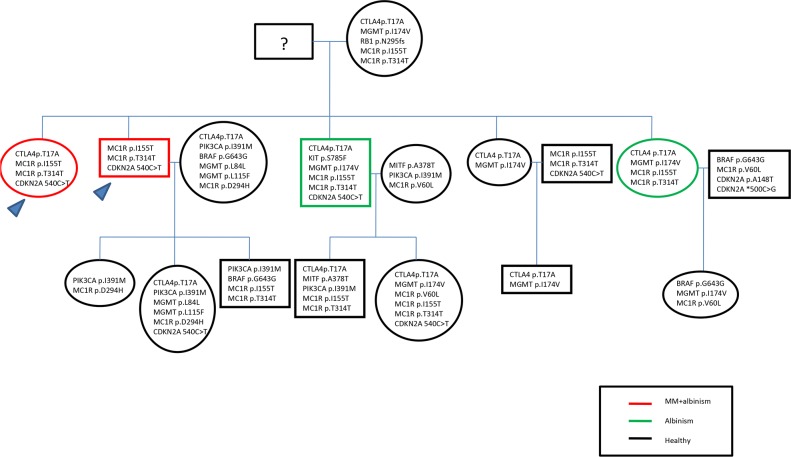
Pedigree including the mutational results

The full length coding sequence and the UTR regions of CDKN2A gene and the Italian founder mutation p.Ser270Asn of POT1 were analyzed by Sanger Sequencing in all indexes and relatives, evidencing two SNPs in 3′UTR region of CDKN2A (c. 500 C>G and c.540C>T) in 41% of all cases. Only one specimen showed a variant known to predispose to melanoma p.Ala148Thr of CDKN2A. Mutational results are summarized in pedigree shown in Figure 1.

### Methylation study

The two index cases and their siblings were investigated for germinal MGMT promoter methylation status. No blood sample resulted to be methylated.

### Prediction, phylogenetic and structural analyses

**Figure 2:**
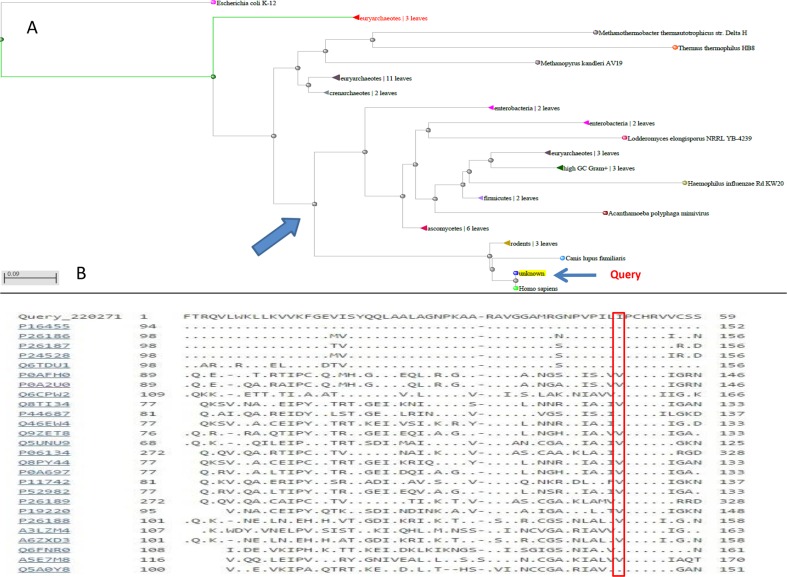
**A**. Dendrogram of the phylogenetic analysis from which the principal node was extracted to show **B**. the alignment of the protein sequences of the most similar species.

**Figure 3:**
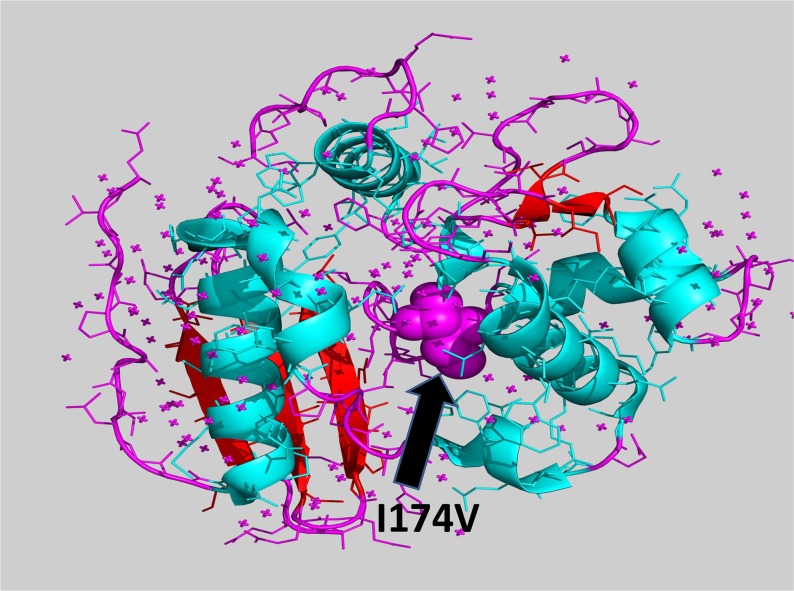
Tridimensional visualization of MGMT p.Ile174Val (I174V)

As MGMT p.Ile174Val alteration was not detected in the index cases, we hypothesized a “protective ” role with respect to melanoma onset of this alteration, primarily to exclude an impairment of the protein function. Annovar predictions were obtained from 5 prediction scores (SIFT, PolyPhen-2, FATHMM, LRT, MutationTaster), 3 conservation scores (GERP++, SiPhy, PhyloP) and 3 ensemble scores (radialSVM, CADD, Condel), as shown in Table 1. All scores but the MutationTaster predicted MGMT p.Ile174Val as a neutral/benign alteration. Phylogenetic analysis of the ATase region (residues 125-204) of MGMT was also performed. In Figure 2A, a dendrogram can be observed from which we selected the principal node including our query to visualize sequence alignments. It was interesting to find that almost all the species carried valine in position 174, supporting results from prediction tools (Figure 2B). Moreover, structural visualization of MGMT p.Ile174Val can be seen in Figure 3. The residue Ile174 lie in a loop between two α-helices, the so-called “Asn-hinge ”, which is very close to the Cys176 in the active site. It is well known that both valine and isoleucine are aliphatic aminoacids, which could be substituted without affecting protein structure. This evidence leads us to consider MGMT p.Ile174Val as a benign alteration.

**Table 1:** Functional annotation of MGMT variants from Annovar results

Annotation tool	Results
SIFT	T
Polyphen2	B
FATHMM	T
LRT	N
MutationTaster	D
RadialSVM	T
GERP++	−0.425
PhyloP	1.655
SiPhy	9.182
Condel	N
CADD	2.658

T: tolerated; B: benign; N: neutral; D: deleterious. Deleterious thresholds: GERP++, score > 4.4; PhyloP, score > 1.6; SiPhy, score > 12.17; CADD, score > 15.

## DISCUSSION

MPM onset is a rare and poorly investigated condition. Family history and the presence of atypical nevi are the two factors most related to MPM. Frequently, CDKN2A and CDK4 are able to explain approximately 50% of fM, and therefore several genes have to be further investigated to better elucidate mechanisms leading to MPM occurrence. Considering the extremely rare coexistence of MPM and OCA, we decided to analyze in the two indexes affected by both conditions, over CDKN2A and CDK4 alterations, a pattern of other genes involved in melanoma predisposition and pathogenesis, in skin/hair pigmentation and in immune pathways. We aimed to individuate possible alterations able to explain the onset of MPM in the OCA indexes, the differences with other three healthy siblings and the possible transmission of these alterations to the relatives.

It was not possible to genetically characterize the grandfather, but neither him nor his wife were affected by albinism. Thus we hypothesized that both of them were healthy carriers of predisposing alterations. In such a context, it was possible to distinguish the genetic pattern of the two indexes affected both by MPM and OCA and of their siblings affected only by the latter. The second generation was also analyzed. In particular, none of the offspring was affected by OCA, which is an autosomal recessive disease. Therefore, as the second generation is unaffected by both pathologies, it was not been considered for further analyses. However, the second generation offspring is under strict follow-up for risk of melanoma.

As regards the most investigated CDKN2A gene, the indexes showed a common SNP in the 3′UTR, c.540 C>T, present in 33% of relatives. No relation of this SNP with melanoma risk has been reported [16]. On the contrary, patients positive for this marker had significantly improved survival [17]. Only one relative showed a CDKN2A variant known as predisposing to melanoma, p.Ala148Thr. Although, this common variant can be considered a polymorphism, it seems to be associated with an increased risk of developing melanoma, probably when present with other modifiers [16].

Another important result of our study concerns the same genetic pattern of the investigated family, probably related to the albinism condition. In fact the indexes and the healthy OCA siblings shared the same MC1R alterations (the strong RHC non synonymous variant p.Ile155Thr and the synonymous alteration p.Thr314=). On the other hand the only sister without MPM and OCA did not show any alteration in MC1R. This gene is a central control point in skin and hair pigmentation. Previous studies reported an association between MC1R and OCA2 variants in determining a modification of the OCA2 phenotype [18,19]. These results evidenced a role of MC1R in OCA occurrence.

Interestingly, our findings revealed a new gene as a probable player in MPM pathogenesis. Indeed, the NGS results evidenced the absence of the MGMT p.Ile174Val variant in the two indexes compared to the other siblings. MGMT was shown to have a crucial role in the DNA repair pathway. The MGMT gene, located in chromosome band 10q26, has previously been associated with several cancer types where its epigenetic silencing has been described, such as glioblastoma, colorectal cancer and gastric cancer [20]. In addition, epigenetic inactivation of MGMT has been demonstrated in melanoma tumor tissue and in the serum of melanoma patients [21,22]. In cancer, MGMT epigenetic silencing confers a poor prognosis, thus it is also an indicator of enhanced responsiveness to treatment with alkylating agents [23]. Moreover, it was also related to familial melanoma [8]. MGMT p.Ile174Val is located in the “Asn-hinge ” portion of the C-terminal domain. The active site-motif (PCHRV) is preceded by two tight turns, stabilized by a highly conserved Asn, which is the core of the “Asn-hinge ”. It contacts the β3 sheet of the N-terminal through the interdomain cleft and is responsible for 40% of the hydrophobic contacts that stabilize the domain interface. Ile174 residue is also involved in such hydrophobic interactions. Both phylogenetic analysis and functional prediction indicated a neutral role for this alteration. Literature regarding p.Ile174Val evidences that this variation has also been called p.Ile143Val due to a different reference sequence [24]. Due to its closeness to the Cys176 active site, population studies have been carried out for different type of cancers. In particular, no effect on melanoma risk was detected [25].

In conclusion, given that the indexes affected by MPM did not carry MGMT p.Ile174Val,and in the light of its neutral role, we hypothesized that the haplotype found in healthy siblings could have a protective role. Moreover, our findings highlighted a possible involvement of MC1R in the occurrence of albinism. The second generation offspring is under strict follow up as some of them have a higher risk of developing multiple primary melanoma according to our model.

## MATERIALS AND METHODS

### Patient information

The index cases were two siblings (a female and a male) affected by both OCA and MPM pathologies.

The female index developed a first primary melanoma in the left leg when she was 57 years old. It was a pT1a superficial spreading melanoma with a Breslow thickness of 0.17 mm, radically removed by surgery. Two years later, she developed a second primary melanoma in the left arm. It was a pT2b ulcerated nodular melanoma with a Breslow thickness of 2 mm associated with metastases in 2 out of 17 left axillar lymphnodes. The genetic analysis of this primary melanoma sample revealed the absence of BRAF mutations, the presence of p.Q61L NRAS mutation and a 40% of somatic MGMT promoter methylation. This index then underwent a regular follow-up. To date, the female index has developed brain and lung metastases and has begun chemo-immunotherapy with a schedule including fotemustine and ipilimumab in an ongoing clinical trial. The male index developed a first primary *in situ* melanoma of the neck region when he was 57 years old, and a second primary nodular melanoma in the right arm four years later. The latest one was a pT1a melanoma with a Breslow thickness of 0.8 mm. Moreover, this index was surgically treated for a basal cell carcinoma and underwent stringent follow-up for the presence of atypical nevi. The primary melanoma sample revealed 11% of somatic MGMT promoter methylation.

Regarding the first generation, the remaining siblings (two females and a male) were enrolled, two of whom had OCA but not melanomas. Moreover, the mother of the indexes, the spouses and the offspring of the entire first generation were enrolled. No relative showed either albinism or melanoma.

The study was approved by the local Ethics Committee of the IRCCS “Giovanni Paolo II” of Bari (prot. no. 515/EC of May 12, 2015) and was performed in accordance with the international standards of Good Clinical Practice. All indexes and their relatives signed informed consent and blood samples from all of them were obtained.

### DNA preparation

DNA was isolated from blood samples using the QIAamp DNA Blood Midi Kit (Qiagen) according to the manufacturer’s instructions. The extracted DNA from each sample was quantified by Nanodrop and Qubit methods.

### Ion torrent PGM library preparation and sequencing

Eleven (BRAF, NRAS, PTEN, MITF, CDK4, MGMT, CTLA4, PIK3CA, MC1R, KIT, RB1) of the 15 genes to investigate were analyzed by a custom panel previously developed by our group [26]. As regards BRCA1/2 analysis we used the Ion AmpliSeq™ BRCA1 and BRCA2 Panel (Termo Fisher Scientific). For both analyses an input of 10 nanograms/each primer pool was required. The experiments were conducted as reported in [26].

### Variant Calling

Data from the PGM runs were processed initially using the Ion Torrent platform-specific pipeline software Torrent Suite to generate sequence reads, trim adapter sequences, and filter and remove poor signal-profile reads. Initial variant calling from the Ion AmpliSeq sequencing data was generated using Torrent Suite Software v5.0 with a plug-in “*variant caller v5.0*” program. In order to eliminate errors in base calling, the Somatic-High Stringency parameters setting was used to generate the final variant calling. Filtered variants were annotated using the Ion Reporter software v5.0 (Termo Fisher Scientific). Mutations were visually examined using Integrative Genomics Viewer (IGV) software (http://www.broadinstitute.org/igv).

Data from the NGS custom panel were also analyzed by a custom pipeline to verify their reliability. In detail, we followed Toolkit for Genome Analysis (GATK, https://www.broadinstitute.org/gatk/) [27] recommendations of DNAseq best practices for calling variants. The following software were used: BWA-mem (http://bio-bwa.sourceforge.net/) for sequence alignment [28] and GATK 3.4 software for the later steps. In detail, after having been aligned to the reference human genome (version hg19), the sequences were not marked for duplicates but underwent realignment around indels, base recalibration and variant discovery (using the haplotype caller function in ERC mode). Since the variant call set was too small (11 genes), hard filters were used to call the gene variants. In detail, the Single Nucleotide Variants (SNVs) were called when the matching the following conditions: GQ (Genotype Quality)≥ 68.5, ReadPosRankSum<-2.42, BaseQRankSum<2.6 and DP>50. Selected variants were functionally annotated by Annovar version 2016Feb01. The LJB* database was used to obtain predictions on deleteriousness from different prediction methods [29,30].

### CDKN2A and POT1 analysis

Mutational screening of exons 1α, 1β, 2 and 3, including the exon-intron boundaries (−34 region, IVS-105, and 3′UTR) of CDKN2A, and of the POT1 region including the p.Ser270Asn mutation, was performed by PCR and direct sequencing on the 3500 Genetic Analyzer (Thermo Fisher Scientific). In detail, PCR amplification of the regions of interest was performed in a Gene-Amp PCR System 9700 (Thermo Fisher Scientific) using the primers listed in Table 2. PCR experiments were performed using: 5 μl of AmpliTaq Gold 360 buffer 10X, 3.33 μl of Magnesium chloride 25 mM, 4 μl of dNTP 2.5 mM, 1 μl of primer forward and reverse 10 μM, 5 μl of GC enhancer and 100 ng of genomic DNA template.

**Table 2:** Sequences of oligos for CDKN2A and POT1 sequencing

Target gene		Sequence (5^’^>3^’^)	Tm [°C]
***CDKN2A***	**1alfa sup_for**	CTCCAGAGGATTTGAGGGAC	59.4
	**1 alfa_rev**	GCGCTACCTGATTCCAATTC	57.3
	**1 alfa_for**	GAAGAAAGAGGAGGGGCTG	58.8
	**1 beta_for**	GGTCCCAGTCTGCAGTTAAG	59.4
	**1beta-art_rev**	GTCTAAGTCGTTGTAACCCG	57.3
	**42F**	GGAAATTGGAAACTGGAAGC	55.3
	**Ex2 inf_rev**	GATTGGCGCGTGAGCTGA	58.2
	**IVS2-105_for**	TGGACCTGGAGCGCTTGA	58.2
	**IVS2-105_rev**	GAAAACTACGAAAGCGGGG	56.7
***POT1***	**POT1_for**	CTATCAGAAGCCCCAGGAAC	59.4
	**POT_1_rev**	ACTGGTCAGTGCCCTCATTG	59.4

PCR amplification conditions were as follows: 95°C for 7 min, 35 cycles of 94°C for 1 min, Tm°C for 1 min, and 72°C for 1 min, followed by a final extension step at 72°C for 7 min.

### MGMT promoter methylation analysis

Promoter methylation status of the MGMT gene was conducted with a PCR-based method using DNA treated with bisulfite followed by a real-time pyrosequencing that targeted CpG islands (EpigenDxInc). This method allowed us to quantify methylation at multiple CpG sites individually. Pyrosequencing was performed using PyroGold Q96 SQA Reagents and the Pyro Q-CpG software on a PyroMark ID pyrosequencer (Qiagen) as per the manufacturer’s recommendation. The sequencing results were analyzed using the PSQ PyroMark software (Qiagen). As controls, CpGenome Universal Methylated DNA (positive methylation control; Chemicon International) and DNA from FFPE non-tumorous skin tissue (negative methylation control) were included in the assay, as well as a reaction without any template DNA (non-template control). All tumour and control specimens were measured in triplicates.

### Phylogenetic and protein structural analyses

The Blastp alignment tool [31] was used to perform a phylogenetic analysis of the MGMT ATase domain. The PyMOL Molecular Graphics System (Version 1.4.1 Schrödinger, LLC) was used to tridimensionally evaluate MGMT p.Ile174Val. PDB IDs for MGMT was 1EH6 [32].

## References

[1] Buljan M, Situm M, Bolanca Z, Zivkovic MV, Mihic LL (2010). Multiple primary melanoma: epidemiological and prognostic implications; analysis of 36 cases. Coll Antropol.

[2] Juul Nielsen L, Rosenkrantz Holmich L (2016). Eleven Primary Melanomas, Colon Cancer, and Atypical Nevi in the Same Patient: A Case Report and Literature Review. Case Rep Dermatol Med.

[3] Palmieri G, Capone M, Ascierto ML, Gentilcore G, Stroncek DF, Casula M, Sini MC, Palla M, Mozzillo N, Ascierto PA (2009). Main roads to melanoma. J Transl Med.

[4] De Giorgi V, Savarese I, D’Errico A, Gori A, Papi F, Colombino M, Sini MC, Stanganelli I, Palmieri G, Massi D (2015). CDKN2A mutations could influence the dermoscopic pattern of presentation of multiple primary melanoma: a clinical dermoscopic genetic study. J Eur Acad Dermatol Venereol.

[5] Puig S, Malvehy J, Badenas C, Ruiz A, Jimenez D, Cuellar F, Azon A, Gonzalez U, Castel T, Campoy A, Herrero J, Marti R, Brunet-Vidal J (2005). Role of the CDKN2A locus in patients with multiple primary melanomas. J Clin Oncol.

[6] Shi J, Yang XR, Ballew B, Rotunno M, Calista D, Fargnoli MC, Ghiorzo P, Bressac-de Paillerets B, Nagore E, Avril MF, Caporaso NE, McMaster ML, Cullen M (2014). Rare missense variants in POT1 predispose to familial cutaneous malignant melanoma. Nat Genet.

[7] Robles-Espinoza CD, Harland M, Ramsay AJ, Aoude LG, Quesada V, Ding Z, Pooley KA, Pritchard AL, Tiffen JC, Petljak M, Palmer JM, Symmons J, Johansson P (2014). POT1 loss-of-function variants predispose to familial melanoma. Nat Genet.

[8] Appelqvist F, Yhr M, Erlandson A, Martinsson T, Enerback C (2014). Deletion of the MGMT gene in familial melanoma. Genes Chromosomes Cancer.

[9] Soura E, Eliades PJ, Shannon K, Stratigos AJ, Tsao H (2016). Hereditary melanoma: Update on syndromes and management: Emerging melanoma cancer complexes and genetic counseling. J Am Acad Dermatol.

[10] Ward KA, Lazovich D, Hordinsky MK (2012). Germline melanoma susceptibility and prognostic genes: a review of the literature. J Am Acad Dermatol.

[11] Read J, Wadt KA, Hayward NK (2016). Melanoma genetics. J Med Genet.

[12] Ghiorzo P, Pastorino L, Queirolo P, Bruno W, Tibiletti MG, Nasti S, Andreotti V, Paillerets BB, Bianchi Scarra G (2013). Prevalence of the E318K MITF germline mutation in Italian melanoma patients: associations with histological subtypes and family cancer history. Pigment Cell Melanoma Res.

[13] Bruno W, Pastorino L, Ghiorzo P, Andreotti V, Martinuzzi C, Menin C, Elefanti L, Stagni C, Vecchiato A, Rodolfo M, Maurichi A, Manoukian S, De Giorgi V (2016). Multiple primary melanomas (MPMs) and criteria for genetic assessment: MultiMEL, a multicenter study of the Italian Melanoma Intergroup. J Am Acad Dermatol.

[14] (1999). Cancer risks in BRCA2 mutation carriers. J Natl Cancer Inst.

[15] Birlea SA, Laberge GS, Procopciuc LM, Fain PR, Spritz RA (2009). CTLA4 and generalized vitiligo: two genetic association studies and a meta-analysis of published data. Pigment Cell Melanoma Res.

[16] Debniak T, Scott RJ, Huzarski T, Byrski T, Rozmiarek A, Debniak B, Zaluga E, Maleszka R, Kladny J, Gorski B, Cybulski C, Gronwald J, Kurzawski G (2005). CDKN2A common variants and their association with melanoma risk: a population-based study. Cancer Res.

[17] Straume O, Smeds J, Kumar R, Hemminki K, Akslen LA (2002). Significant impact of promoter hypermethylation and the 540 C>T polymorphism of CDKN2A in cutaneous melanoma of the vertical growth phase. Am J Pathol.

[18] King RA, Willaert RK, Schmidt RM, Pietsch J, Savage S, Brott MJ, Fryer JP, Summers CG, Oetting WS (2003). MC1R mutations modify the classic phenotype of oculocutaneous albinism type 2 (OCA2). Am J Hum Genet.

[19] Preising MN, Forster H, Gonser M, Lorenz B (2011). Screening of TYR, OCA2, GPR143, and MC1R in patients with congenital nystagmus, macular hypoplasia, and fundus hypopigmentation indicating albinism. Mol Vis.

[20] Paska AV, Hudler P (2015). Aberrant methylation patterns in cancer: a clinical view. Biochem Med (Zagreb).

[21] Marini A, Mirmohammadsadegh A, Nambiar S, Gustrau A, Ruzicka T, Hengge UR (2006). Epigenetic inactivation of tumor suppressor genes in serum of patients with cutaneous melanoma. J Invest Dermatol.

[22] Rastetter M, Schagdarsurengin U, Lahtz C, Fiedler E, Marsch W, Dammann R, Helmbold P (2007). Frequent intra-tumoural heterogeneity of promoter hypermethylation in malignant melanoma. Histol Histopathol.

[23] Soejima H, Zhao W, Mukai T (2005). Epigenetic silencing of the MGMT gene in cancer. Biochem Cell Biol.

[24] Deng C, Xie D, Capasso H, Zhao Y, Wang LD, Hong JY (1999). Genetic polymorphism of human O6-alkylguanine-DNA alkyltransferase: identification of a missense variation in the active site region. Pharmacogenetics.

[25] Egyházi S, Ma S, Smoczynski K, Hansson J, Platz A, Ringborg U (2002). Novel O6-methylguanine-DNA methyltransferase SNPs: a frequency comparison of patients with familial melanoma and healthy individuals in Sweden. Hum Mutat.

[26] Pinto R, De Summa S, Strippoli S, Pilato B, Azzariti A, Guida G, Guida M, Tommasi S (2016). The next generation of metastatic melanoma: Uncovering the genetic variants for anti-BRAF therapy response. Oncotarget.

[27] McKenna A, Hanna M, Banks E, Sivachenko A, Cibulskis K, Kernytsky A, Garimella K, Altshuler D, Gabriel S, Daly M, DePristo MA (2010). The Genome Analysis Toolkit: a MapReduce framework for analyzing next-generation DNA sequencing data. Genome Res.

[28] Li H, Durbin R (2009). Fast and accurate short read alignment with Burrows-Wheeler transform. Bioinformatics.

[29] Yang H, Wang K (2015). Genomic variant annotation and prioritization with ANNOVAR and wANNOVAR. Nat Protoc.

[30] Dong C, Wei P, Jian X, Gibbs R, Boerwinkle E, Wang K, Liu X (2015). Comparison and integration of deleteriousness prediction methods for nonsynonymous SNVs in whole exome sequencing studies. Hum Mol Genet.

[31] Altschul SF, Madden TL, Schäffer AA, Zhang J, Zhang Z, Miller W, Lipman DJ (1997). Gapped BLAST and PSI-BLAST: a new generation of protein database search programs. Nucleic Acids Res.

[32] Daniels DS, Mol CD, Arvai AS, Kanugula S, Pegg AE, Tainer JA (2000). Active and alkylated human AGT structures: a novel zinc site, inhibitor and extrahelical base binding. EMBO J.

